# Whole Genome Sequencing of an Unusual Serotype of Shiga Toxin–producing *Escherichia coli*

**DOI:** 10.3201/eid1908.130016

**Published:** 2013-08

**Authors:** Tim Dallman, Lisa Cross, Chloe Bishop, Neil Perry, Bente Olesen, Kathie A. Grant, Claire Jenkins

**Affiliations:** Health Protection Agency, London, UK (T. Dallman, L. Cross, C. Bishop, N. Perry, K.A. Grant, C. Jenkins);; Hillerød Hospital, Hillerød, Denmark (B. Olesen)

**Keywords:** Escherichia coli, Shiga toxin–producing Escherichia coli, bacteria, pathogenicity, whole genome sequencing, gene transfer, serotype, O117:K1:H7

## Abstract

Shiga toxin–producing *Escherichia coli* serotype O117:K1:H7 is a cause of persistent diarrhea in travelers to tropical locations. Whole genome sequencing identified genetic mechanisms involved in the pathoadaptive phenotype. Sequencing also identified toxin and putative adherence genes flanked by sequences indicating horizontal gene transfer from *Shigella dysenteriae* and *Salmonella* spp., respectively.

There are >400 serotypes of Shiga toxin–producing *Escherichia coli* (STEC), and >100 of these are known to be associated with severe disease in humans (*1*). STEC are defined by the presence of 1 or both phage-encoded Shiga toxin genes *stx1* and *stx2*. However, those serotypes associated with more severe disease generally harbor additional virulence genes, such as *eae* (intimin), which is encoded on the locus of enterocyte effacement, or virulence regulation genes, such as *agg*R, which is located on the aggregative adherence plasmid. Both of these genes mediate attachment of the bacteria to the host gut mucosa (*2*). The *stx*1 gene is also found in *Shigella dysenteriae* serotype 1.

A range of molecular typing methods show that the shigellae belong within the *Escherichia coli* species (*3*). Peng et al. (*4*) described an evolutionary path of *Shigella* spp. from *E. coli* involving gene acquisition (virulence plasmid and pathogenicity islands) and gene loss (pathoadaptivity). Gene loss, or loss of gene function, may result from changes to bacterial biosynthesis pathways driven by the abundance of resources in the host or because the genes may encode proteins adverse to bacterial virulence.

Olesen et al. (*5*) described a strain of STEC serotype O117:K1:H7 found in travelers from Denmark who returned from tropical locations. The strain was unusual because it was negative for the production of lysine decarboxylase and β-galactosidase (ortho-nitrophenol test) and positive only for *stx*1.

Since 2004, 19 isolates of STEC O117:K1:H7 have been submitted to the Gastrointestinal Bacteria Reference Unit at the Health Protection Agency in London, UK, from frontline diagnostic microbiology laboratories in England and Wales for confirmation of identification and typing (Table). All isolates were originally misidentified by the submitting laboratory as *Shigella sonnei* or *Shigella* spp., probably because of the unusual biochemical phenotype exhibited by this strain. The purpose of this study was to use whole genome sequencing to investigate the evolutionary origins, putative virulence genes, and pathoadaptive mechanisms of this unusual STEC serotype.

**Table:** Shiga toxin–producing *Escherichia coli* O117:K1:H7 strains submitted to GBRU from frontline diagnostic microbiology laboratories, United Kingdom, 2004–2012*

Strains	Year isolated (no.)	Clinical signs and symptoms of patient	Country or region visited by patient
Strains sequenced			
151/06	2006	Not reported	India
371/08	2008	Not reported	Egypt
290/10	2010	Bloody diarrhea	Cuba
754/10	2010	Pyrexia	South America
229/11	2011	Diarrhea, abdominal pain	Kenya and India
Additional strains	2004 (2); 2008 and 2009 (3); 2010 (4); 2011 (2); 2012 (2)	Diarrhea, bloody diarrhea, fever, persistent nausea, abdominal pain	Afghanistan, Bali, Egypt, Ecuador, Ghana, India, Jordan, Libya, Tanzania, Uganda

*GBRU, Gastrointestinal Bacteria Reference Unit.

## The Study

DNA from 5 isolates (151/06, 371/08, 290/10, 754/10, and 229/11) was prepared for sequencing by using the Nextera sample preparation method and sequenced with a standard 2 × 151 base protocol on a MiSeq instrument (Illumina, San Diego, CA, USA) (*6*). Sequences were analyzed as described (*7*). In brief, Velvet version 1.1.04 (www.ebi.ac.uk/~zerbino/velvet/) was used to produce an average of 489 contigs with an average N50 length of 38722. Illumina reads were mapped to the reference strain (GenBank accession no. CU928145) by using Bowtie2 2.0.0 β-5 (http://bowtie-bio.sourceforge.net/bowtie2/) and a variant call format file was created from each of the binary alignment maps, which were further parsed to extract only single nucleotide polymorphism (SNP) positions that were of high quality in all genomes.

Concatenated SNPs generated against the reference strain 55989 were used to produce a maximum-likelihood phylogeny of 5 strains in the Gastrointestinal Bacteria Reference Unit archive and 36 other publically available *E. coli* genomes and *Shigella* spp. (Figure). Despite temporal and spatial diversity of the 5 sequenced isolates, they clustered on the same branch, but they were distant from other publically available sequences of STEC strains.

**Figure:**
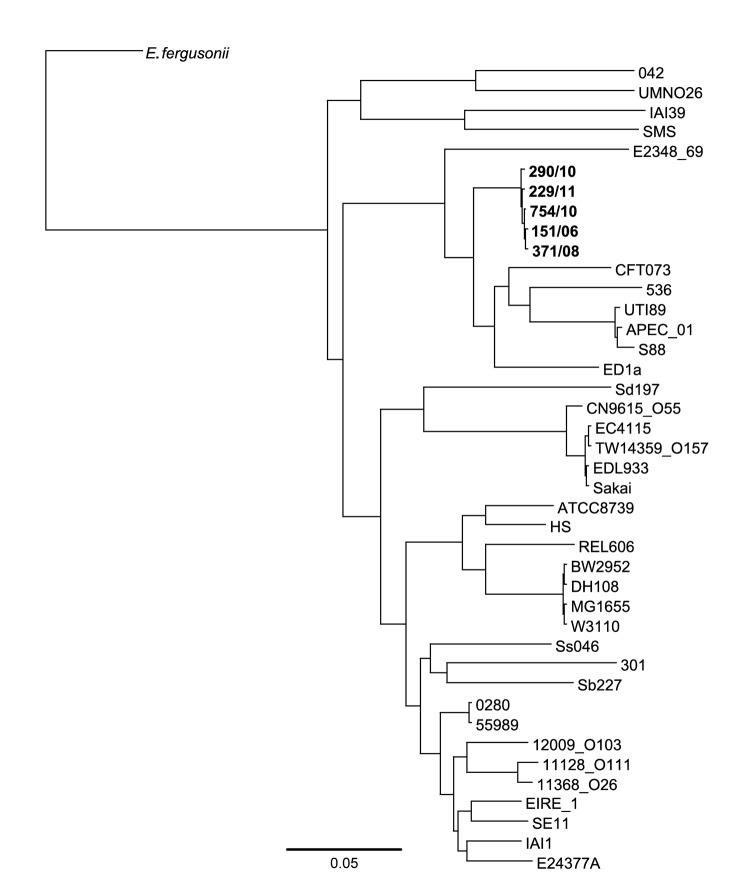
Maximum-likelihood dendrogram for 5 strains of Shiga toxin–producing *Escherichia coli* serotype O117 in the Gastrointestinal Bacteria Reference Unit (Health Protection Agency, London, UK) archive (**boldface**), 32 other *E. coli* genomes, and 4 *Shigella* spp. genomes. *E. fergusonii* was used as an outgroup. Scale bar indicates nucleotide substitutions per site.

A phylogenetic tree based on a diverse range of *E. coli* showed that the 5 strains of STEC O117 have 130 polymorphic positions, and the closest 2 strains (299/11 and 754/10) are 26 SNPs apart (Table; Figure). Furthermore, on the basis of a diverse range of *E. coli*, genome sequences of EDL933 and Sakai, 2 well-described strains of STEC O157, are ≈35 SNPs apart. The multilocus sequence type ST504 was assigned in accordance with the *E. coli* multilocus sequence type databases at the Environment Research Institute, University College (Cork, Ireland).

## Conclusions

Alignment of the genome of strain 229/11 with STEC O157 (EDL933) and *Shigella dystenteriae* serotype 1 (Sd197) indicated gene acquisition, loss, and rearrangement in 229/11. The *stx*1 gene is adjacent to the *yjh*S gene in 229/11 and Sd197, and in 229/11 this fragment is flanked by phage-like sequences that are closely related to Stx2-converting phage sequences but not to other Stx1-converting phages. This unusual gene arrangement was described by Sato et al. (*8*). In Sd197, this region is flanked by integrases and insertion sequences. Other open reading frames homologous to those of *Shigella* spp. in *stx*-flanking regions in *E. coli* have been described, and it is likely that *E. coli* and the shigellae have exchanged *stx* many times in their evolutionary past but only certain strains, such as 229/11, have the appropriate genomic background to retain and stably express Stx (*9*).

Strain 229/11 also contains a 10-kb pathogenicity island (PAI) harboring the *rat*A, *Siv*l, and *Siv*H genes and shares homology with PAI CS54 found in *Salmonella* spp. (*10*) and a PAI found in avian pathogenic *E. coli* (*11*). *Siv*H has been described as similar to the intimin gene (*10*). *Siv*H may facilitate attachment to the host gut mucosa and could explain the long persistence of STEC O117:K1:H7 in infected patients (*5*). In vitro inactivation of *sivH* in *S. enterica* serovar Typhimurium resulted in a reduced ability to colonize Peyer’s patches (*10*). In *S. enterica* serovar Typhimurium, CS54 is 25-kb and encodes *shd*A, *rat*A, *rat*B, *siv*l, and *siv*H, whereas in *S. enterica* subsp. II, *S. bongori* serotypes and 229/11, *rat*B, and *shd*A are absent (*10*).

Cadaverine has an inhibitory effect on enterotoxin activity by preventing full expression of the virulent phenotype, and it has been suggested that there is evolutionary pressure to mutate or delete the *cadA* gene (*12*). This gene is missing from *S. flexneri* (Sf301) and *S. boydii* (Sb227) because of inversion-associated deletions, and in Sd197 and *S. sonnei* (Ss046) it is inactivated by a frameshift mutation and an insertion sequence, respectively (*12*). In 229/11, loss of *cad*A (lysine decarboxylase) activity is caused by repositioning of the of the *cad*A activator gene, *Cad*C, upstream of the *cad*A gene and a 90-bp deletion at the 5′ end of *cad*C. The *cad*A gene and truncated *cad*C gene are separated by a large fragment of DNA inserted into the *cad*C gene. This fragment contains several open reading frames, including genes encoding aerobactin siderophore biosynthesis proteins.

Lactose fermentation is a biochemical property commonly used for distinguishing *Shigella* spp. from *E. coli* because shigellae are non- or late-lactose fermenters. In Sd197 and Ss046 (late lactose–fermenting strains), the key gene, *lac*Z (encoding β-d-galactosidase) is intact, although *lac*Y (encoding galactose permease) is a pseudogene (*12*). Like Sf301 and Sb227, *lac*Z and *lac*Y are deleted in strain 229/11. The lack of a functional *lac* operon has been associated with pathogenicity mechanisms in *S. enterica* (*13*).

*E. coli* as a species contains a large diversity of adaptive paths. This diversity is the result of a highly dynamic genome, with a constant and frequent flux of insertions and deletions (*3*). Pathogenicity in STEC O117:K1:H7 is most likely multifactorial and results from a novel combination of lack of *cad*A and *lac*Z expression and the presence of *stx*1 and the intimin-like *siv*H genes, demonstrating pathoadaptivity and horizontal gene transfer.
